# Endothelial protein C receptor is overexpressed in colorectal cancer as a result of amplification and hypomethylation of chromosome 20q

**DOI:** 10.1002/cjp2.70

**Published:** 2017-07-14

**Authors:** Neeraj Lal, Carrie R Willcox, Andrew Beggs, Philippe Taniere, Aarti Shikotra, Peter Bradding, Richard Adams, David Fisher, Gary Middleton, Chris Tselepis, Benjamin E Willcox

**Affiliations:** ^1^ Cancer Immunology and Immunotherapy Centre, Institute of Immunology and Immunotherapy University of Birmingham Edgbaston, Birmingham UK; ^2^ Institute of Cancer and Genomic Sciences University of Birmingham Edgbaston, Birmingham UK; ^3^ Department of Histopathology Queen Elizabeth Hospital Birmingham, Mindelsohn Way Edgbaston, Birmingham UK; ^4^ Department of Infection, Immunity and Inflammation, Institute for Lung Health University of Leicester Leicester UK; ^5^ Institute of Cancer & Genetics Cardiff University School of Medicine, Velindre Hospital Cardiff UK; ^6^ MRC Clinical Trials Unit University College London London UK

**Keywords:** automated microscopy, cell culture, colon, digital image analysis, digital microscopy, *in vitro* models, neoplasia

## Abstract

Endothelial Protein C Receptor (EPCR) is a Major Histocompatibility Complex homologue, with established roles downregulating coagulation and in endothelial protection. Expressed predominantly on endothelium, EPCR affects inflammatory, apoptotic and cell proliferation pathways by binding to activated protein C (APC). However, EPCR can also be expressed on cancer cells, although the underlying reasons are unclear. Moreover, although EPCR has been linked with chemosensitivity in lung cancer, its clinical significance in many tumours is unknown. Here, we explored its significance in colorectal cancer (CRC). Bioinformatic methods revealed EPCR overexpression in many epithelial cancers, which was confirmed on CRC epithelial tumour cells by immunohistochemistry. EPCR upregulation resulted from gene amplification and DNA hypomethylation, and occurred in concert with a cohort of neighbouring genes on chromosome 20q, a region previously implicated in chemoresistance. As in endothelial cells, EPCR reproducibly mediated ERK pathway activation in a model CRC cell line following APC treatment. However, EPCR knockdown studies failed to highlight compelling EPCR‐intrinsic impact on CRC cell phenotype, with limited effects on chemosensitivity and no effect on invasion observed, while EPCR appeared to decrease CRC cell migration. Consistent with these observations, differential EPCR expression did not influence response to chemotherapy in a human CRC cohort. Our results provide a compelling explanation for how EPCR is upregulated in diverse epithelial malignancies. They indicate that the clinical significance of EPCR varies across different tumour types. Furthermore, they raise the possibility that the prognostic significance of EPCR in certain tumours relates significantly to co‐upregulation of neighbouring genes on chromosome 20q. Therefore, efforts to exploit EPCR as a prognostic marker should be focussed on specific tumours, and in such scenarios EPCR‐co‐dysregulated genes may represent potential axes for therapeutic intervention.

## Introduction

Endothelial protein C receptor (EPCR) is a type I transmembrane protein largely restricted in expression to endothelium. Homologous to Major Histocompatibility Complex molecules, it has well recognised roles in dampening coagulation, and in endothelial protection, which are initiated via its interaction with activated Protein C 1, 2, 3, 4, 5. There has been increasing interest in EPCR's potential role and clinical significance in cancer, following several reports indicating overexpression on epithelial tumour cells 6, 7. However, studies in different tumour types 8, 9, 10, 11, 12, largely focused on exploring EPCR‐intrinsic effects on cancer cell phenotype or tumour progression in murine models, have yielded conflicting results regarding the effects of EPCR on epithelial tumourigenesis. EPCR expression in *in vitro* systems and mouse models has been proposed to increase tumour cell proliferation/migration 2, or increases metastatic burden 1, in gastric and lung cancer, respectively. In a murine breast cancer model EPCR distinguished a cancer stem cell‐like population with a high tumour‐initiating capacity, and *in vivo* EPCR blockade attenuated tumour growth 10. Conversely, in murine models of melanoma 4, and mesothelioma 5 EPCR expression decreased metastasis, limiting tumour growth and burden, respectively.

Despite such conflicting results, EPCR overexpression in cancer clearly may be clinically relevant. EPCR was found to be a marker of chemoresistance in tumour cell lines 6, including colorectal cancer cell lines such as HCT116. Furthermore, EPCR expression is predictive for chemotherapy response in early stage non‐small cell lung cancer 8. Finally, in ovarian cancer, serum EPCR expression correlates with the tumour marker CA‐125, suggesting possible clinical relevance as a biomarker 13.

Here we examined the overexpression of EPCR in cancer, focusing on its role in colorectal cancer (CRC). This stemmed from our previous work highlighting EPCR as a direct ligand for Vδ2‐negative γδ T cells 14, 15, which are the predominant tissue subset of these unconventional T cells, and are thought to possess potent anti‐tumour effector capabilities. We sought to understand the extent and significance of EPCR expression in epithelial cancers, including the cellular mechanisms underlying its overexpression, its functional significance in transformed tumour cells and its clinical significance. Notably, EPCR‐associated signalling pathways in endothelium have potential relevance in cancer, overlapping with key proliferative (ERK/AKT) and apoptotic pathways (BAX, BCL2), and raising the possibility that dysregulated ECPR expression on transformed epithelial tissue may directly effect similar mechanisms to promote tumour cell survival and growth. The role of EPCR in CRC, a tumour type with high mortality and prevalence, has not been explored, and in view of the well‐established developmental pathway and pathological characterisation in this setting, CRC was selected as a promising human model in which to clarify the role of EPCR in tumourigenesis.

## Methods

### TCGA bioinformatic analysis

Oncomine (Compendia Bioscience, Thermo Fisher Scientific, Waltham, Massaschusetts, USA) was used for analysis and visualisation of EPCR expression in multiple tumour types 16. EPCR mutation, methylation, copy number, expression data, and pathological and clinical data were extracted from The Cancer Genome Atlas (TCGA) project via the cBioportal tool 17, 18, 19, 20. Cancer cell line encyclopaedia (CCLE) 21 data were extracted from the CCLE portal and cBioportal. Data were tabulated and analysed with The Integrative Genomics Viewer 22 and Excel (Microsoft Corp., Redmond, Washington, USA). Pearson correlations were performed for parametric data, and Spearman correlations for nonparametric data. Significance tests were performed in Minitab (Minitab Inc., State College, Pennsylvania, USA) using T‐tests for parametric data and Mann‐Whitney U for nonparametric data. Normality of distributions were confirmed using the Anderson–Darling Test.

### Sample collection

Patient tumour specimens used to confirm EPCR expression were collected from the University of Birmingham Human Biomaterials Resource Centre (HBRC) (approval number: 11‐058). Survival analysis used tumour samples from the MRC COIN study (ISRCTN27286448), under ethical approval 13/WM/0339 23. All patients had provided informed consent for tissue usage.

### Immunohistochemistry

Immunohistochemistry (IHC) was performed as previously reported 24 (supplementary materials and methods). IHC slides were imaged as whole slides on a Vectra 2.0 (Perkin Elmer) imaging system. Subsequent imaging analysis used inForm software (Perkin Elmer, Waltham, Massaschusetts, USA), and involved development of trained tissue and cell segmentation algorithms validated by a consultant pathologist (PT). Immunoreactivity intensities were determined on a per cell basis, and H scores (the product of reactivity intensity [0–3] and percentage coverage [0–100] giving a score from 0–300) were created by the inForm software. Tumour region and Stroma region H scores across all slides were collated and compared using the unpaired T‐test.

COIN study slides were stained for EPCR at the University of Birmingham's HBRC biobank using a Bondmax Autostainer (Leica Biosystems, Wetzlar, Germany). Anti‐EPCR primary antibody (R&D Systems, Minneapolis, Minnesota, USA, clone 304519) was incubated for 10 minutes (dilution of 1:200) with antigen retrieval at pH 9. Whole slides were scanned using a Leica SCN400 (Leica Biosystems, Wetzlar, Germany) slide scanner. Scanned slides were analysed using Definiens Tissue Studio software (Definiens AG, Munich, Germany). Tumour regions of each slide were manually identified, and trained segmentation algorithms used to separate epithelium and stromal regions. EPCR immunoreactivity was then analysed on a regional basis, by automated quantitation of the percentage of pixels within tense, moderate, weak or no staining in each area, and used to create a percental score for each region. Hypermutation status data were available for tumour samples from the COIN samples, allowing comparison with staining results. The majority of the samples analysed were microsatellite stable (MSS) (103/153), whereas the remainder either did not have data or failed microsatellite testing. No statistically significant correlation with EPCR staining level was observed.

Clinico‐pathological data for both the local and COIN trial cohort of CRC cases are presented in supplementary material, Tables S1 and S2, respectively.

### shRNA transfection

Five EPCR shRNA clones and one control shRNA lentiviral particles were obtained from Sigma‐Aldrich (St Louis, Missouri, USA) (supplementary materials and methods). HCT116 cells or HT29 cells were grown to 70–80% confluence in Dulbecco's Modified Eagle Medium (DMEM) with Fetal Calf Serum (FCS). shRNA lentiviral particles were added at multiplicity of infection (MOI) of 5, 1.5 and 0.5. shRNA transductants were selected for Puromycin (Sigma‐Aldrich, St Louis, Missouri, USA) resistance. EPCR‐overexpressing HT29 cells were a kind gift from Julie Déchanet‐Merville (University of Bordeaux, France).

### Activated protein C ERK phosphorylation

Activated Protein C (APC, Sigma‐Aldrich, St Louis, Missouri, USA) was added to confluent HCT116 cells after 48 h of serum starvation. APC was added in the presence or absence of EPCR function blocking antibodies after which cells were lysed. Cell lysates were separated by gel electrophoresis, transferred to PVDF, and probed with anti‐ERK/pERK primary antibodies (New England Biolabs, Ipswich, Massachusetts, USA, 4370S &4695S) and goat anti‐rabbit HRP conjugated secondary antibodies, prior to development using ECL.

### Microarray

HCT116 cells were treated with APC after serum starvation (supplementary materials and methods). RNA was extracted from cells using the RNeasy Plus Kit (Qiagen, Hilden, Germany) alongside negative controls. Two‐colour Agilent Microarray was carried out at the Functional Genomics Facility (University of Birmingham); three experimental replicates and a minimum of two technical replicates were performed for each specimen. APC treated cells were assigned to Cy5 and controls to Cy3. Differential gene expression data were produced in R using the Limma package (Bioconductor) after ‘Loess’ normalisation 25, 26, 27. Genes with a Bayes factor >5 or an adjusted *P* value < 0.05 (Benjamini and Hochberg's method) were considered for further analysis, which was performed using DAVID 28, 29 and GSEA 30, 31.

### MTT, BrdU, and migration assays

Established protocols were used to carry out 3‐(4,5‐dimethylthiazol‐2‐yl)‐2,5‐diphenyltetrazolium bromide (MTT) metabolic activity assays and bromodeoxyuridine (BrdU) cell proliferation assays [following manufacturer's instructions, Roche, (Roche Applied Science, Penzberg, Germany)] as previously described 32 and migration assays in wild type and EPCR knockdown cells (see supplementary materials and methods).

### Survival analysis

Clinical data for patients in the COIN study were obtained from the MRC Clinical Trials Unit (supplementary materials and methods). The median EPCR percentage score was used to divide patients into EPCR high and low categories. Additionally, a separate analysis with COIN data was carried out to compare the top 20% of EPCR expressors against the bottom 20%. Statistical modelling was performed using Stata 12.1 (Stata Corp, College Station, Texas, USA). Both prognostic and predictive analyses utilized the Cox proportional hazards model, and adjusted for factors that significantly associated with progression free survival (PFS) to *p* <0.05. Given that baseline 1‐year survival was ∼24%, if the comparator group had a higher response than this, 153 samples had 80% power to detect an increase in 1‐year survival from 24 to 45% (HR = 0.56), and 70% power to detect an increase to 42% (HR = 0.61). If the comparator group had a *lower* response, 153 samples had 80% power to detect a decrease in 1‐year survival from 24 to 9.5% (HR = 1.66), and 70% power to detect a decrease to 11% (HR = 1.57). The top/bottom 20% of EPCR expressors were separately compared, with cut offs for EPCR expression based on median expression of EPCR, thereby dividing patients into two equal groups. No significant differences were observed.

## Results

### EPCR is overexpressed in multiple tumour types

The Oncomine database was interrogated to compare EPCR mRNA expression in multiple tumour types relative to normal tissue. EPCR was upregulated in tumour versus normal in 126 separate datasets and downregulated in 50 datasets, which included 21 different cancer types. Of all cancer types, CRC had the highest level of EPCR overexpression, and the most datasets in total in which EPCR was overexpressed (Table 1). In CRC, across nine separate nonoverlapping datasets, EPCR was strikingly overexpressed (*p* < 0.0001). This suggested that amongst all tumours, CRC had one of the highest and most consistent levels of EPCR mRNA upregulation.

**Table 1:** ONCOMINE expression data – number of datasets in which EPCR is significantly differentially expressed in cancer versus normal to *p* < 0.0001. Cell colour is determined by the highest overexpression (left column) or under expression (right column) gene rank percentile for EPCR

Analysis type by cancer	Cancer versus normal	
Bladder cancer	4	
Brain and CNS cancer	13	
Breast cancer	3	9
Cervical cancer	3	
Colorectal cancer	22	
Oesophageal cancer	8	2
Gastric cancer	8	
Head and Neck cancer	14	
Kidney cancer	9	1
Leukaemia	8	1
Liver cancer	4	2
Lung cancer		5
Lymphoma	11	1
Melanoma		2
Myeloma	1	
Other cancer	12	4
Ovarian cancer		10
Pancreatic cancer	5	
Prostate cancer		9
Sarcoma	2	4

Percentile of under expressed genes containing EPCR			Percentile of overexpressed genes containing EPCR		
1%	5%	10%	10%	5%	1%
					

### EPCR is expressed in colorectal cancer

**Figure 1:**
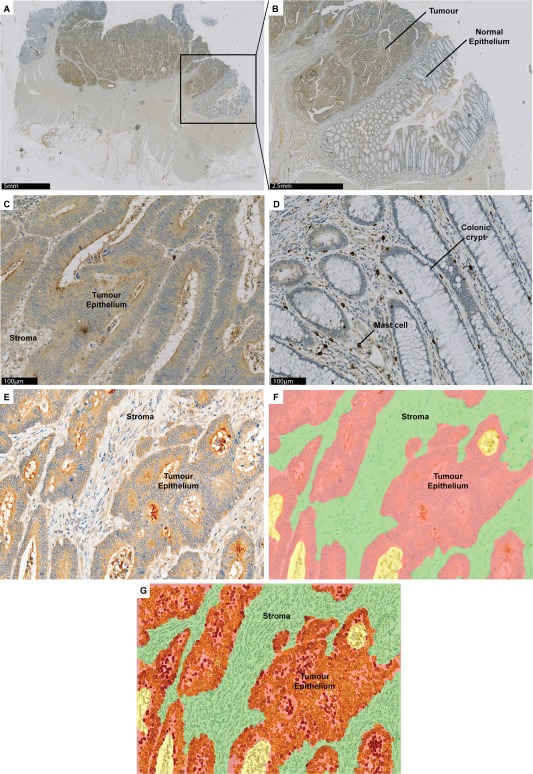
Digital software analysis of EPCR‐stained CRC sections confirms that EPCR is overexpressed on tumour compared to normal epithelium. (A) Whole slide scanned image of CRC region surrounded by normal colonic mucosa. (B) 5x magnified image of tumour with adjacent normal colonic mucosa. (C) 20× magnified image of tumour region, demonstrating strong EPCR staining. (D) 20× magnified image of normal region, with negative staining of colonic crypts. (E) Area for digital analysis with inForm software. (F) Image (E) with inForm tissue‐segmentation through automated algorithm. Red = epithelium, green = stroma, yellow = background. (G) Image (F) with staining intensity within tumour cells. Red = strong, orange = moderate, yellow = weak.

To confirm upregulation in CRC at a protein level, EPCR expression was assessed in whole sections from 30 colorectal cancers and adjacent normal regions using immunohistochemistry (IHC). Before this, EPCR immunoreactivity was validated by EPCR knockdown in HCT116 cells, with staining of endothelial vessels a further positive control in normal/tumour sections (supplementary material, Figure S1). Overall, the majority of immunoreactivity was observed in tumour epithelial tissue. Sections were imaged on the Vectra platform and EPCR immunoreactivity assessed using inForm software (Figure 1). This confirmed that EPCR expression was higher in tumour regions compared to normal regions (*p* < 0.0001, mean H scores 244.2 and 87.6, respectively). In addition, samples from the COIN clinical trial were used to independently confirm EPCR expression in CRC (*n* = 153). Immunoreactivity was assessed using Definiens Tissue Studio, allowing independent confirmation of EPCR expression in terms of both the cohort examined and the digital pathology scoring package used. Positive EPCR staining was observed in all cases. In epithelium, the mean H score was 216.1 (range 155.2 – 296.0). In stroma, the mean H score was 211.5 (range 180.7 – 261.4).

Finally, immunoreactivity to what appeared to be mast cells was observed consistently across all tissue sections, and confirmed by flow cytometry and immunoreactivity to individual cells with both EPCR‐specific mAb and mast cell marker mAbs in serial sections (supplementary material, Figure S2). This is the first report of immunoreactivity to EPCR on mast cells to our knowledge.

### EPCR overexpression in CRC is mediated by gene amplification and hypomethylation

**Figure 2:**
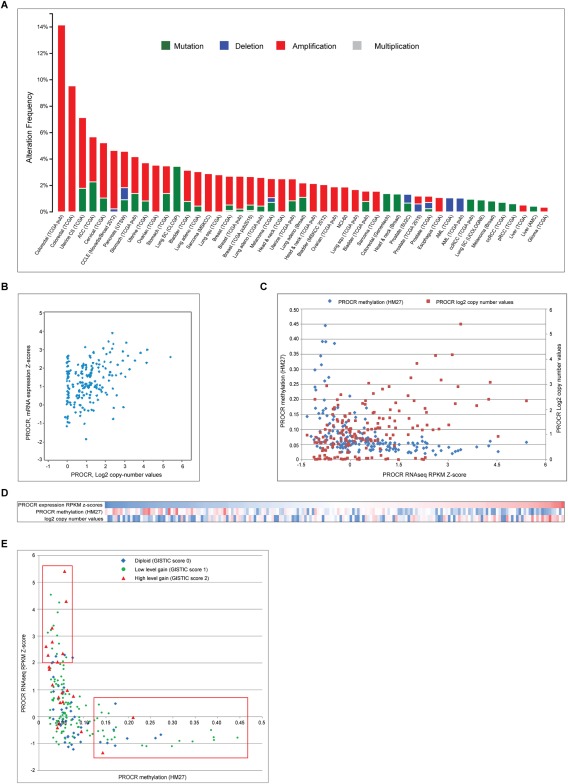
EPCR expression is associated with *PROCR* gene amplification and hypomethylation. (A) The EPCR gene (*PROCR*) is frequently amplified but rarely deleted or mutated across a range of cancer types and databases 17, 20. (B) In colon cancer, increases in *PROCR* copy number are associated with higher mRNA expression (*p* <0.01). (C) Copy number and methylation both impact upon EPCR expression. This graph includes 195 colorectal cancer patients from the TCGA dataset. The highest EPCR expression is in patients with amplification of *PROCR* in association with low methylation. Lower expression is commonly associated with higher methylation and diploid copy numbers or low level gain. (D) Heatmap showing correlation between *PROCR* mRNA expression, promoter methylation and copy number values (blue = low, red=high). EPCR correlates positively with amplification and negatively with methylation. (E) Relationship between *PROCR* methylation, GISTIC copy number scores and expression. The upper box highlights the group with low methylation and highest expression. These patients tend to have low level gain or high level amplification. The lower box highlights most highly methylated patients, who have low expression regardless of copy number status.

To determine possible mechanisms underpinning EPCR overexpression, we analysed public bioinformatic genomic and transcriptomic datasets 19. The EPCR gene (*PROCR*), though rarely mutated in any cancer type, was frequently amplified (Figure 2A). In the CRC TCGA dataset, 73.8% of tumours had either low level gain or high level amplification of the *PROCR* gene [GISTIC (Genomic Identification of Significant Targets in Cancer) score of 1 (low level amplification) or 2 (high level amplification)]. In CRC, gene amplification was significantly associated with increased mRNA expression (*p* <0.05). Overall, there was a strong correlation between *PROCR* copy number and mRNA expression (Figure 2B), (Spearman rho = 0.325, *p* < 0.0001). Of the top 10% of EPCR expressors, 90% had either low level gain (GISTIC score 1) or high level amplification (GISTIC score 2) of the *PROCR* gene. Moreover, of the top 10%, two cancers were GISTIC 0 whereas seven were GISTIC 2; conversely, of the bottom 10%, seven were GISTIC 0 whereas two were GISTIC 2 (*p* = 0.06). Using the same dataset, chromosomally unstable (CIN) cancers had significantly higher EPCR expression than the non‐CIN group (z‐scores of 0.448 and 0.030, respectively, *p* < 0.01). Despite this, some EPCR over expressors were of the non‐CIN microsatellite unstable (MSI) hypermutated subtype. These tumours were mostly diploid; thus, gene amplification could not account for overexpression. We therefore suspected that epigenetic factors might also influence EPCR expression. Indeed, there was a strong inverse correlation between *PROCR* promoter methylation and gene expression across the entire patient dataset (Spearman rho −0.59, *p* < 0.001). Overall, 92.8% of CRCs were hypomethylated at the *PROCR* promoter (HM27 < 0.2). Of the top 10% of EPCR expressors (*N* = 20), all were hypomethylated at this promoter (β‐value < 0.2), whereas of the bottom 10% (*N* = 20), five were hypermethylated (β‐value < 0.2; *p* = 0.047). When copy number data were combined with methylation data, it became clear that hypomethylation played a key role in determining expression across all patient groups (Figure 2C,D). Tumours overexpressing EPCR had significantly lower methylation than those that did not (*p* < 0.001). Amplification and hypomethylation when combined associated with the highest expression (Figure 2E).

### EPCR expression is associated with chromosome 20q amplification

**Figure 3:**
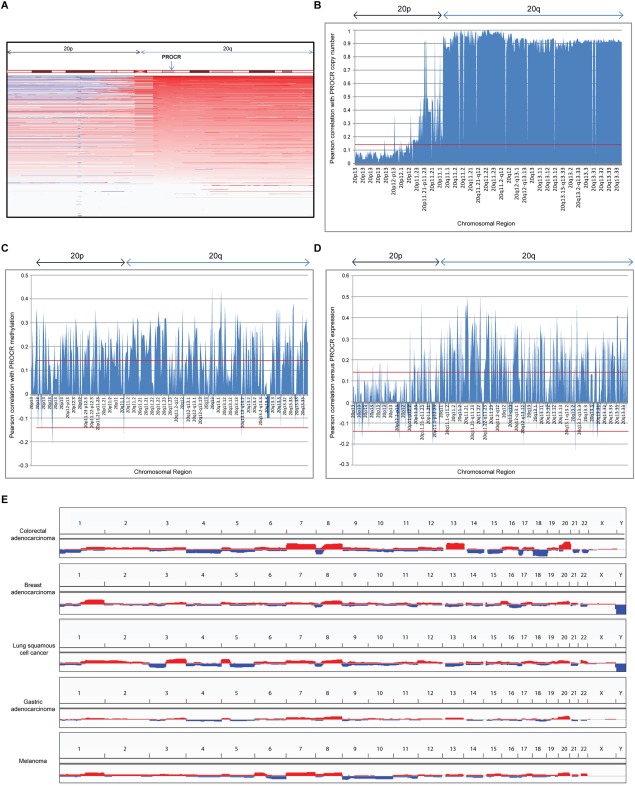
EPCR expression is associated with chromosome 20q amplification. (A) Copy numbers of chromosome 20 genes in 195 TCGA colorectal cancer patients. Genes are organised by chromosomal location (20p is left, 20q is right). Blue represents loss of gene, red represents gain of gene and white represents no change in copy number. The chromosome 20q region is commonly amplified in CRC 22. The location of *PROCR* is indicated by the arrow. (B‐D) Pearson correlations for *PROCR* gene copy number (B), methylation (C), and expression (D) with genes on chromosome 20, arranged by chromosome region. *PROCR* is located on 20q11.2. Red lines represent the thresholds of statistical significance (Pearson value > 0.1405 or <−0.1405, significant to *p* <0.05). (E) Chromosome 20q is one of the most frequently amplified regions in colon cancer and across a range of cancer types (colon adenocarcinoma, breast adenocarcinoma, lung squamous cell cancer, gastric adenocarcinoma and melanoma TCGA data). Chromosomes are ordered from 1 to 23, X, Y. Images (A) and (E) were created using Integrated Genomics Viewer and Cbioportal.

To determine whether *PROCR* was co‐expressed with other genes, we explored TCGA expression and genetic data. Most genes whose expression correlated most closely with *PROCR* were located in the same chromosomal region as *PROCR* – chromosome 20q (Table 2). On an individual patient basis, *PROCR* gene amplification was frequently associated with amplification of all genes on chromosome 20q (Figure 3A). Across the entire 195 patient cohort, the copy numbers of most (99.42%) chromosome 20q genes correlated positively with *PROCR* copy number (Figure 3B), whereas a significantly smaller proportion of 20p genes were correlated (36.90%, *p* < 0.05 [chi‐squared]). Also, 55.33% of chromosome 20q genes correlated with *PROCR* in terms of promoter methylation. However, a similar proportion of chromosome 20p genes were also correlated in terms of methylation (51.30%, *p* = 0.47, chi‐squared; Figure 3C). Finally, the expression of a high proportion (53.80%) of chromosome 20q genes correlated positively with *PROCR* expression, although the correlations were not as large in magnitude as those seen with copy number (Figure 3D). A significantly smaller proportion of chromosome 20p genes were correlated in terms of expression (13.72%, *p* < 0.05 [chi‐squared]). This suggests that whilst *PROCR* copy number and expression co‐regulation are a chromosome 20q regional phenomenon, regulation of methylation is less region specific.

**Table 2:** Genes most highly co‐expressed with EPCR in TCGA CRC dataset, and their chromosomal locations

Gene Symbol	Spearman Score versus PROCR	Chromosomal location
HM13	0.45	20q11.21
PDRG1	0.44	20q11.21
C20ORF24	0.43	20q11.23
PSMA7	0.43	20q13.33
TPD52L2	0.43	20q13.33
TRPC4AP	0.43	20q11.22
MRGBP	0.43	20q13.33
ADRM1	0.42	20q13.33
BRI3	0.42	7q21
EIF6	0.41	20q11.22
ATP6V1F	0.41	7q32.1
EFNA2	0.41	19p13.3
SSUH2	0.41	3p25.3
NDRG3	0.4	20q11.23
RHOD	0.4	11q13.2
EDEM2	0.39	20q11.22
ACTR5	0.39	20q11.23
DYNLRB1	0.39	20q11.22
CEBPA	0.39	19q13.11
FAM96B	0.39	16q22.1
UQCC1	0.38	20q11.22
PIGU	0.38	20q11.22
ROMO1	0.38	20q11.22
AHCY	0.38	20q11.22
NEU1	0.38	6p21.33
TOMM34	0.38	20q13.12
TCFL5	0.38	20q13.33
RPS21	0.38	20q13.33
TLDC2	0.37	20q11.23

Across the entire colon cancer dataset, chromosome 20q was amongst the most frequently amplified chromosomal regions (Figure 3E). Additionally, chromosome 20q was amplified across a range of cancer types, including several in which EPCR has been shown to be expressed, including melanoma 11, gastric cancer 9 and lung squamous cell cancer 8 (Figure 3E). These data suggest that EPCR may be a marker of tumours with chromosome 20q amplification, which has been linked with poor outcome 33, 34.

Carvalho *et al* 34 previously identified three regions of chromosome 20q commonly amplified in CRC 34. *PROCR* is located within the first of these (SRO1), which spans 32–36 Mb. The group identified seven putative oncogenes based on upregulation in carcinomas versus adenomas, association with chromosome 20q amplification, and correlation between copy number and gene expression. Of these seven genes, six correlated significantly with EPCR expression (Table 3). Furthermore, EPCR expression was correlated significantly with 13/13 ‘cancer initiating genes’ located on chromosome 20q identified by Tabach *et al* 33. Finally, *PROCR* correlated significantly with a gene ranked first in a microarray‐based CIN signature (TPX2) 35, reinforcing the view that its expression is associated with chromosomal instability.

**Table 3:** Correlation of Carvalho *et al*'s putative oncogenes 34 located on chromosome 20q versus *PROCR*. Correlation values show Pearson scores (R) versus *PROCR* in the TCGA CRC dataset. *p* < 0.05 where *R* > 0.1405, highlighted red

	Chromosomal location	Copy number	Methylation	Expression
C20orf24	20q11.22	0.989164	0.227405	0.498511
AURKA	20q13.2	0.907967	0.009653	0.239549
RBM38 (RNPC1)	20q13.32	0.928319	0.21619	0.053815
NELFCD (TH1L)	20q13	0.901242	0.155924	0.334379
ADRM1	20q13.33	0.920162	0.295276	0.454516
MRGBP (C20orf20)	20q13.33	0.909783	N/A	0.409151
TCFL5	20q13.3‐qter	0.909783	0.214221	0.364161

Having established that EPCR upregulation denotes regional dysregulation, we then sought to test the significance of upregulated EPCR expression on CRC tissue.

### EPCR is expressed on multiple CRC cell lines

First, we investigated EPCR expression in CRC cell lines. Using the Cancer Cell Line Encyclopaedia 21, we selected five lines with high EPCR mRNA expression (HCT116, HT29, RKO, COLO320 and SW480), and confirmed EPCR protein expression with flow cytometry (supplementary material, Figure S3A–E). Both HCT116 and HT29 had high expression, and these were selected for further *in vitro* studies as they represent the two main biological subtypes of CRC (MSI‐H and CIN respectively). EPCR expression was also investigated in the non‐tumourigenic AA/C1 adenoma cell line 36. AA/C1 expressed low levels of EPCR. However, a tumourigenic derivative (AA/C1 10C) had increased EPCR expression (supplementary material, Figure S3F,G).

### Exogenous activated protein C (APC) induces EPCR‐dependent ERK phosphorylation in CRC lines

**Figure 4:**
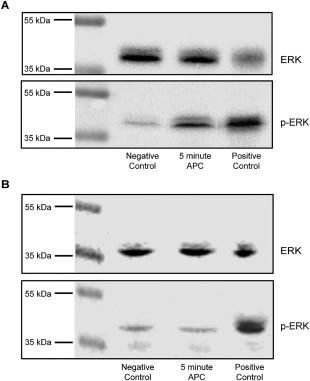
APC induces ERK phosphorylation, which is inhibited by EPCR‐blockade. (A) Western blotting for pERK and Total‐ERK of lysates from serum‐starved HCT116 cells that had been treated with APC for 5 min. Negative controls were serum starved only. Positive controls were treated with 50% FCS for 10 min. (B) Western blotting after pre‐treatment with EPCR‐blocking antibody.

APC treatment of vascular endothelial cells has been shown to induce EPCR‐dependent activation of ERK pathways, which are implicated in epithelial transformation 37. To establish a proof of principle that exogenous APC could also induce ERK phosphorylation in a model CRC cell line, we treated serum‐starved HCT116 cells with APC for 5 min. This led to an increase in ERK phosphorylation (Figure 4A), which was inhibited by EPCR‐blocking antibodies (Figure 4B), confirming that such APC‐induced ERK phosphorylation was EPCR dependent. In addition, to determine if CRC cells were capable of producing endogenous Protein C (PC), serum‐starved HCT116 cells were lysed and western blots performed for PC (supplementary material, Figure S4A). PC was detected in the cell lysate, and protease‐activated receptor 1 (PAR1) staining was observed alongside EPCR in sections from identical blocks (supplementary material, Figure S4B,C). These results establish that EPCR can in principle activate ERK signalling when expressed on CRC cells in response to APC, and that components of the APC pathway can be expressed in CRC *in vivo*.

### APC treatment affects gene transcription in HCT116 cells

Having established that APC can induce ERK phosphorylation in HCT116 cells, the effect of APC treatment on gene transcription was then assessed. APC treatment of serum‐starved HCT116 cells and subsequent microarray analysis identified a set of differentially expressed genes (Bayes factor > 5) (supplementary material, Table S3).

Gene set enrichment analysis (GSEA) (Table 4 and supplementary material, Figure S5) revealed that a large proportion of genes were ribosomal and/or associated with gene transcription; and also, that several gene sets associated with epidermal growth factor (EGF) signalling were enriched (supplementary material, Figure S5A–C), consistent with ERK pathway stimulation. Finally, a gene set containing genes upregulated by thrombin signalling in HUVEC cells was enriched, consistent with thrombin and APC's common signalling pathways (supplementary material, Figure S5D).

**Table 4:** Top 30 gene sets in gene set enrichment analysis of APC‐treated HCT116 cells versus control cells

Name of gene set	Size (Genes)	Enrichmen*t* score	FDR q value	FWER *P* value	Rank at max
REACTOME_PEPTIDE_CHAIN_ELONGATION	83	0.830811	0	0	958
REACTOME_INFLUENZA_VIRAL_RNA_TRANSCRIPTION_AND_REPLICATION	97	0.791672	0	0	958
REACTOME_SRP_DEPENDENT_COTRANSLATIONAL_PROTEIN_TARGETING_TO_MEMBRANE	106	0.767984	0	0	958
KEGG_RIBOSOME	85	0.838541	0	0	958
REACTOME_3_UTR_MEDIATED_TRANSLATIONAL_REGULATION	91	0.791225	0	0	958
REACTOME_NONSENSE_MEDIATED_DECAY_ENHANCED_BY_THE_EXON_JUNCTION_COMPLEX	102	0.748643	0	0	958
REACTOME_TRANSLATION	131	0.695274	0	0	958
REACTOME_INFLUENZA_LIFE_CYCLE	130	0.687707	0	0	958
BILANGES_SERUM_AND_RAPAMYCIN_SENSITIVE_GENES	61	0.72625	0	0	958
REACTOME_FORMATION_OF_THE_TERNARY_COMPLEX_AND_SUBSEQUENTLY_THE_43S_COMPLEX	35	0.815359	0	0	958
REACTOME_ACTIVATION_OF_THE_MRNA_UPON_BINDING_OF_THE_CAP_BINDING_COMPLEX_AND_EIFS_AND_SUBSEQUENT_BINDING_TO_43S	43	0.737235	0	0	958
CHNG_MULTIPLE_MYELOMA_HYPERPLOID_UP	44	0.714276	0	0	958
REACTOME_METABOLISM_OF_MRNA	202	0.517838	0	0	958
FLOTHO_PEDIATRIC_ALL_THERAPY_RESPONSE_UP	50	0.642381	0	0	1173
NAGASHIMA_EGF_SIGNALING_UP	52	0.629965	0	0	2671
PECE_MAMMARY_STEM_CELL_DN	128	0.529283	0	0	1489
REACTOME_METABOLISM_OF_RNA	245	0.453754	0	0	958
NAGASHIMA_NRG1_SIGNALING_UP	160	0.485804	7.41E‐05	0.001	2099
CHASSOT_SKIN_WOUND	10	0.878359	2.08E‐04	0.003	1642
JECHLINGER_EPITHELIAL_TO_MESENCHYMAL_TRANSITION_DN	63	0.526858	3.91E‐04	0.006	886
HOLLEMAN_ASPARAGINASE_RESISTANCE_B_ALL_UP	22	0.674004	3.73E‐04	0.006	551
REACTOME_OLFACTORY_SIGNALING_PATHWAY	102	0.474621	5.87E‐04	0.01	3844
REACTOME_METABOLISM_OF_PROTEINS	382	0.395476	8.40E‐04	0.015	2516
UZONYI_RESPONSE_TO_LEUKOTRIENE_AND_THROMBIN	34	0.597898	8.05E‐04	0.015	4451
TIEN_INTESTINE_PROBIOTICS_6HR_UP	50	0.533304	0.00148	0.028	1092
HSIAO_HOUSEKEEPING_GENES	363	0.389684	0.00247	0.049	1536
LEE_LIVER_CANCER_HEPATOBLAST	15	0.701492	0.00248	0.051	3202
AMIT_EGF_RESPONSE_40_HELA	39	0.545263	0.00395	0.083	2970
YAMASHITA_LIVER_CANCER_WITH_EPCAM_UP	46	0.535978	0.00390	0.085	1692
BILANGES_SERUM_RESPONSE_TRANSLATION	33	0.579625	0.00436	0.099	1489

### EPCR knockdown increases chemoresistance and migration in HCT116 cells

**Figure 5:**
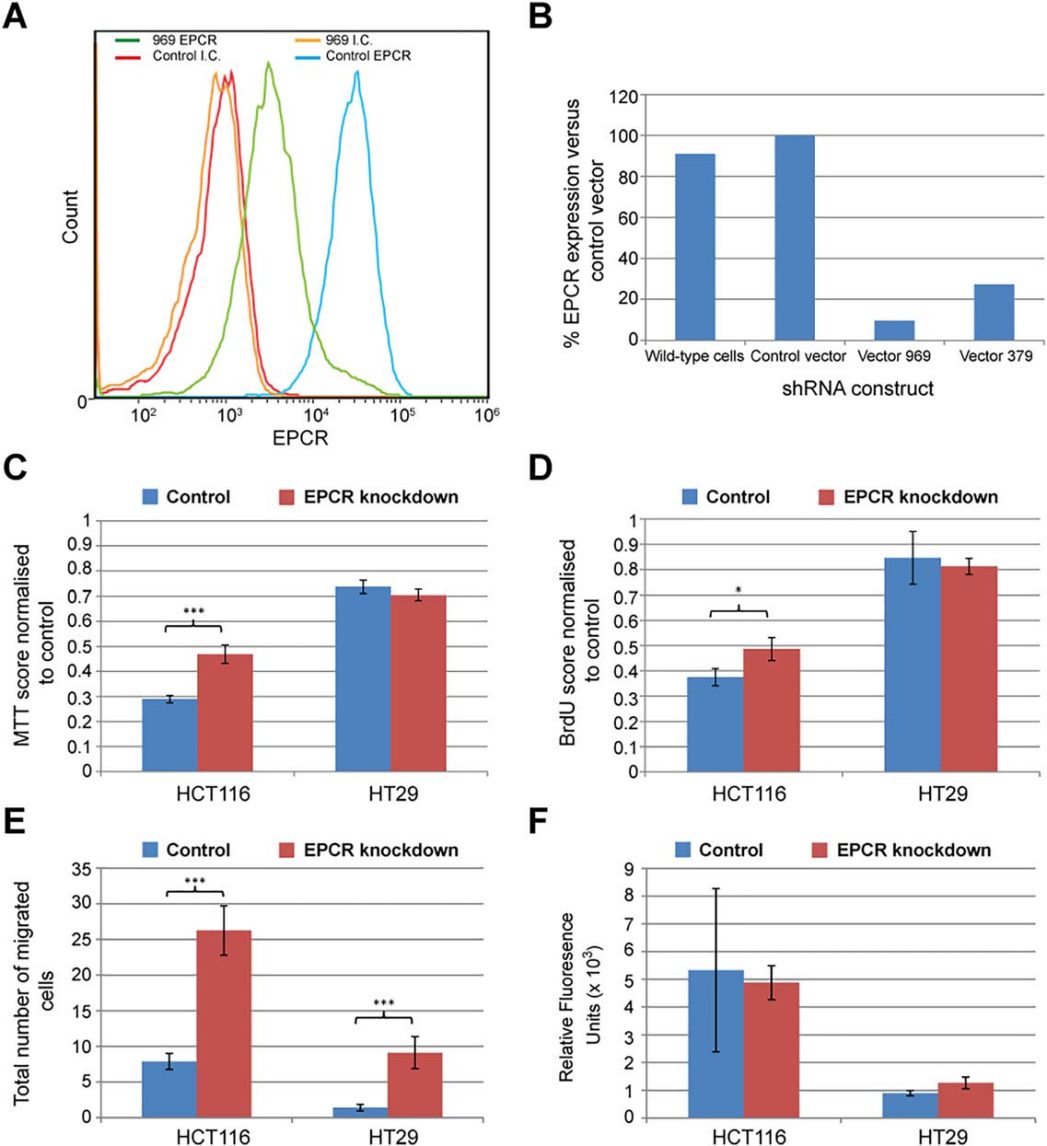
In HCT116 cells, EPCR knockdown with shRNA decreases chemosensitivity and increases migration. (A) Flow cytometry data confirming knockdown of EPCR expression with shRNA (vector 969) compared to control cells. (B) EPCR expression in wild‐type cells and shRNA transfected cells (vectors 969 and 379), as a percentage of control vector‐transfected cells. (C) MTT scores for control and shRNA knockdown (clone 969) HCT116 and HT29 cells after treatment with 5FU (32 μM), shown as a percentage of control cells. (D) BrdU scores for control and shRNA knockdown (clone 969) HCT116 and HT29 cells after treatment with 5FU (32 μM), shown as a percentage of control cells. (E) 48 h Transwell migration assay – number of cells that migrated through Transwell membrane for each clone in five counted regions. (F) 48 h QCM invasion assay. In panels (C‐F), asterisks represent statistical significance of EPCR‐low cells versus control cells (*p < 0.05, **p <0.01, ***p <0.001). Bars represent standard error.

Having established that EPCR signalling can induce ERK phosphorylation and alter gene transcription, we aimed to determine whether EPCR overexpression provided any functional benefit to tumour cells. Previous work has demonstrated that EPCR is a marker of chemoresistant cell lines, including HCT116 6. To further examine the effect of EPCR expression on chemosensitivity, we knocked down EPCR expression in HCT116 cells using two shRNA constructs, which induced high (clone 969 – 90%) and medium (clone 379 – 60%) levels of knockdown. EPCR knockdown significantly reduced the toxicity of 5FU and epirubicin in MTT and BrdU assays of cellular viability and proliferation (Figure 5A–D), suggesting EPCR increased chemosensitivity in this setting.

Transwell assays were performed to determine how EPCR expression affected cellular migration. Over 48 hours, HCT116 cells with high EPCR knockdown had a significantly higher rate of migration than control cells (*p* < 0.001) or medium‐level EPCR knockdown cells (*p* < 0.001) (Figure 5E). However, no significant differences between the groups were observed in invasion assays.

To determine whether the effect of EPCR perturbation was similar in a MSS/CIN cell line, we repeated experiments in HT29 cells. In HT29 cells, EPCR knockdown did not consistently affect MTT and BrdU chemosensitivity assays. However, as with HCT116 cells, high EPCR knockdown (>95%) increased cellular migration in the Transwell assay (*p* < 0.01). No difference was observed with knockdown in QCM invasion assays. HT29 EPCR overexpression (to over sixfold above wild type HT29) did not consistently affect chemosensitivity, migration, or invasion.

### EPCR expression is not predictive for chemotherapy or cetuximab responsiveness in CRC

Previous studies have indicated that EPCR is a marker for chemoresistant cell lines 6, and conversely its expression may predict chemotherapy responses in early stage lung cancer 8. Furthermore, we have shown that EPCR perturbation can marginally affect CRC cell line chemosensitivity. We wanted to determine whether EPCR could affect chemosensivity *in vivo*. In addition, having established that EPCR can mediate APC‐dependent ERK phosphorylation on CRC cells, we were also interested in the potential impact of EPCR upregulation on clinical responses to EGFR monoclonal antibodies (mAb) in CRC patients. Patients with *KRAS* mutation do not respond to EGFR mAbs due to ‘bypass signalling’ resulting from constitutively active MEK/ERK signalling. As EPCR‐mediated signalling induces ERK phosphorylation, we hypothesised that EPCR could also act as a bypass signalling pathway in an analogous manner.

**Figure 6:**
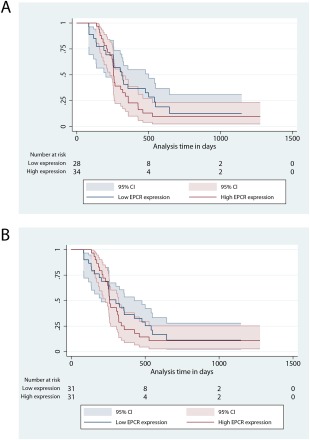
Kaplan‐Meier curves for progression free survival in EPCR high and low patients, divided by median EPCR expression in Tumour epithelium (A) and Tumour stroma (B). The blue lines represent low EPCR expression, and the red lines represent high EPCR expression. This cohort contains 153 patients from the MRC COIN trial.

To determine whether differential EPCR expression is associated with altered chemotherapy or EGFR monoclonal antibody (cetuximab) responsiveness in CRC patients, immunohistochemistry for EPCR was performed on 153 CRC tumour samples from the MRC COIN study 23 and analysed using Definiens Tissue Studio software (see supplementary materials and methods). Of these, 71 were from the chemotherapy arm, and 82 were from the chemotherapy plus cetuximab arm. Survival analyses indicated no significant difference in PFS between EPCR high and low cases (divided by median EPCR expression) across all patients, irrespective of whether it was assessed on tumour epithelium or stroma (Figure 6, *p* = 0.367 and 0.568, respectively), when both treatment arms were analysed individually, or when comparing the top and bottom 20% of EPCR expressors. Finally, EPCR did not predict for survival in either treatment arm after exclusion of *KRAS* mutant cases. These data suggest that, given the power of the analysis (see Methods section), the extent of EPCR expression does not predict for chemotherapy or cetuximab responsiveness in CRC.

## Discussion

EPCR, a receptor with antiapoptotic and proliferative effects 3, 4, 5, is known to be expressed on various cancer cell lines 6, 38. However, its expression in CRC has not previously been addressed. Using robust digital immunohistochemistry algorithms and bioinformatic analyses we have demonstrated that EPCR is aberrantly expressed in CRC tissue, with expression increased in cancer compared to normal mucosa in all cases tested. Of note, the use of a robust quantitative, pathologist‐informed digital pathology algorithm (via the InForm software package) that was specifically educated to analyse colonic tissue enabled statistically significant EPCR upregulation to be detected in a relatively small sample set (30 in‐house samples). Confirmation of this upregulation via a comparable but independent approach and algorithm using the Definiens Tissue Studio package, and applied to a separate sample set, provided powerful corroboration that EPCR is indeed aberrantly upregulated at the protein level in CRC.

In addition, although EPCR expression has been investigated in various epithelial cancers, the mechanisms underlying its upregulation have remained unclear. Importantly, our study shines light on these. We show that EPCR upregulation results from both chromosome 20q amplification and promoter hypomethylation, processes likely relevant to multiple tumour types. Specifically, chromosome 20q amplification occurs as a result of chromosomal instability (due to loss of the CIN suppressor genes of chromosome 18q 39) and occurs in many cancer types 33, 34, 40, 41, 42. Of relevance, previous studies have confirmed EPCR expression in breast cancer, lung cancer and melanoma, but these studies have not related EPCR expression to chromosomal amplification or hypomethylation 8, 10, 11. Significantly, all these tumour types are associated with chromosome 20q amplification.

We then investigated the implications of aberrant EPCR expression on CRC cell functional phenotype, following studies highlighting EPCR effects on cancer cell migration, invasion and proliferation in different tumour settings 8, 11, 12, 38. While our results establish the proof of principle that EPCR, via APC binding, can stimulate ERK signalling and elicit changes in gene transcription (as in endothelial cells), a number of limitations are worth considering. First, an important caveat is that these analyses were carried out on a single cancer cell line (HCT116); clearly future studies could address how the effects of APC compared in different cellular contexts. Second, how these effects might be modulated by the genetics of *in vivo* tumourigenesis is unclear, as is whether APC can be produced in sufficient quantities to elicit the EPCR‐mediated effects we observed. Finally, how the diverse spectrum of tumour microenvironmental factors might influence such effects is unclear. Nevertheless, this *in vitro* result at least establishes that, in principle, APC exposure to EPCR‐overexpressing cancer cells has the potential to influence cancer cell signalling and transcription. Despite these findings, our *in vitro* data failed to identify compelling evidence that overexpression of EPCR *per se* might provide functional advantages for CRC cells. Interestingly, in both HCT116 and HT29 cells, EPCR expression appeared to decrease migration in *in vitro* assays. Furthermore, EPCR had variable effects on cancer cell phenotype across cell lines:specifically, it increased the chemosensitivity of HCT116, consistent with a report that higher EPCR expression was associated with superior chemotherapy response in early stage lung cancer 8. In contrast, no chemosensitivity effects were observed in HT29 cells, and no effects on invasion were observed in either cell line. However, an important limitation of these experiments, and of approaches highlighting both beneficial and detrimental effects of EPCR perturbation in animal models 8, 9, 11, 12, is that they primarily address EPCR‐intrinsic effects on cancer development, and typically focus on a small number of cell lines. Our findings underline that the effects of EPCR upregulation on cancer cells may be heavily dependent on biological context, including the exact cancer cell line (even within a single cancer cell type) and potentially upregulation of functionally important neighbouring genes co‐amplified on chromosome 20q. Indeed, while the reasons underlying aberrant EPCR expression on the different cancer cell lines we studied was not explored (eg chromosome 20q amplification versus promoter hypomethylation), it might conceivably affect the nature of any co‐expressed genes, and contribute to the diverse results observed. This is a potential focus for future studies. This perspective demonstrates the need for models that represent the variety of biological variants observed in the clinical scenario.

Our data establish that EPCR expression should be interpreted in the context of chromosome 20q amplification, which is itself associated with an aggressive and invasive phenotype, tumour progression and metastasis formation 8, 33, 34, 38, and is suggested to play a causative rather than a bystander role in tumour progression 33. Despite this and previous reports highlighting links between EPCR and chemoresistance/chemosensitivity 6, 8, analysis of samples from the MRC COIN study did not reveal any association between EPCR expression and PFS in advanced CRC patients treated with chemotherapy, indicating that the impact of the EPCR‐high phenotype was insufficient to predict chemosensitivity in this setting, although we cannot exclude the possibility of relatively subtle effects beyond the power of our analysis. Also, as EPCR mediates ERK phosphorylation in CRC cells, we hypothesised it could act as a bypass pathway during EGFR inhibition with cetuximab, similar to *KRAS* mutation 23, and might represent an additional negative predictive biomarker of cetuximab response. However, survival analysis of EPCR‐stained specimens from the COIN trial also failed to show any association with PFS in the cetuximab treatment arm, although formally we cannot exclude the possibility of smaller effects, beyond the power of our analysis to detect.

These negative results may reflect that EPCR upregulation occurs too early in tumourigenesis to be a useful discriminator of clinical outcome following diagnosis of late stage CRC. Consistent with this, chromosome 20q amplification is absent in normal colonic mucosa, but increases as disease advances from non‐progressed adenomas to progressed adenomas, and is found in most metastatic samples 34, 40, 43. This is consistent with our findings that AA/C1 10C, a malignant variant of the AA/C1 adenoma cell line, had increased EPCR expression 36, and our observation that all CRC samples expressed EPCR to some extent. Also, our analyses highlighted that *PROCR* expression correlated strongly with 6/7 putative oncogenes and 13/13 ‘cancer initiating genes’, all located alongside it on chromosome 20q 33, 34. In CRC, aberrant EPCR expression may therefore result from chromosome 20q amplification occurring early in transformation, which is suggested to promote cancer initiation independently of other chromosomal abnormalities 33. In contrast, the finding that high EPCR expression predicts for positive chemotherapeutic response in lung cancer 8 suggests that it may represent a clinically useful biomarker in some settings. Although this could reflect EPCR‐intrinsic effects on cancer cell phenotype, the effect of regionally co‐expressed genes could alternatively explain the EPCR‐high phenotype, and EPCR may represent a clinically useful surrogate marker of chromosome 20q amplification in this setting. This possibility justifies further investigation, but could imply an impact of chromosome 20q genes on chemosensitivity. Owing to its relevance in dampening coagulation, evaluation of EPCR as a biomarker of thrombotic risk in cancer is also of interest.

Previously we highlighted EPCR as a ligand for human γδ T‐cell receptors (TCRs), mediating recognition of CMV‐infected endothelial cells by Vδ2‐negative T cells 15. This study suggests that EPCR in principle could act as stress ligand for γδ T cells in cancer, as it will be consistently upregulated in multiple transformed tissues, including in early stages of tumourigenesis. Clearly further work is required to determine whether such aberrant EPCR expression may induce γδ T cell responses in epithelial tumours.

## Author contributions statement

NL, CW: designed the study, carried out experiments, analysed data, and wrote the manuscript. AB: assisted with bioinformatics analyses and survival analyses. PT: assisted with immunohistochemistry procedures and digital pathology algorithm validation. AS and PB: planned and carried out studies on EPCR expression in mast cells. GM, RA, DF: helped design EPCR staining and linked survival analysis of COIN samples. CT, BW: designed the study, analysed data and wrote the manuscript.

*Cited only in supplementary material.

## Supporting information

SUPPLEMENTARY MATERIAL ONLINE

**Supplementary materials and methods**

**Supplementary figure legends**

**Figure S1.** Validation of EPCR antibody. (A) IHC staining of wild‐type HCT116 cells with anti‐EPCR antibody. (B) IHC staining of EPCR shRNA knockdown HCT116 cells with anti‐EPCR antibody. (C) Positive staining of endothelium

**Figure S2.** EPCR expression in mast cells. (A) Flow cytometry demonstrating EPCR protein expression on mast cells. (B) Flow cytometry data are presented for the HMC‐1 cell line and human lung mast cells (HLMCs) as percentage of 2E9+ cells of the total cell population using the Overton method (47)(HLMC n=3). (C‐D) Sequential sections demonstrating co‐localisation of ECPR to tryptase positive mast cells within airway tissue (black arrows ×200 magnification). HM

**Figure S3.** Expression of EPCR on colorectal cancer cell lines. Expression of EPCR on (A) Colo320, (B) RKO, (C) SW480, (D) HT29, and (E) HCT116 cells by flow cytometry. Expression of EPCR on (F) AA/C1 non‐tumourigenic adenoma cells or (G) the tumourigenic AA/C1/10C derivative. Isotype control staining is shown in grey and EPCR staining in black

**Figure S4.** Detection of EPCR‐associated proteins in CRC. (A) Detection of Protein C in HCT116 cell lysate. The anti‐Protein C antibody detects both Protein C (62 KDa) and Activated Protein C (APC, 21 KDa). Exogenous APC was used as a positive control. A faint band was detected in the 60‐65KDa range in the HCT116 lysate, suggestive of the presence of Protein C. (B) and (C) PAR1 immunohistochemistry in (B) CRC and (C) normal colon

**Figure S5.** Gene set enrichment analyses. Enrichment plots for gene sets: Nagashima EGF signalling up (A), Zwang EGF persistently up (B), Amit EGF response 40 Hela (C), Uzonyi response to leukotriene and thrombin (D). Lower panels – all genes ranked in order of most overexpression after APC treatment to most underexpression. 0 represents equal expression after treatment. Middle panels ‐ the black vertical bars represent genes from the relevant gene set. Top panels ‐ the green line represents the running enrichment score for the gene set as the analysis walks down the ranked list, increasing the running‐sum statistic when a gene is in a gene set and decreasing it when it is not. As the peaks of these green lines are towards the overexpression side of the gene list, these gene sets are highly enriched

**Table S1.** Clinico‐pathological data for the local colorectal cancer cohort

**Table S2.** Clinico‐pathological data for the COIN trial colorectal cancer cohort

**Table S3.** Genes differentially expressed with APC treatment of HCT116 cells (Bayes Factor >5). Log FC shows fold change (base 2) in APC treated cells versus controls. 0 therefore represents equal expression in the APC treated and control cells
