# Supplementary material for: Endothelial protein C receptor is overexpressed in colorectal cancer as a result of amplification and hypomethylation of chromosome 20q
Source: J Pathol Clin Res. 2017 Jul 14;3(3):155–70. doi: 10.1002/cjp2.70 (PMC5527318; doi:10.1002/cjp2.70)
Supplement: Supplementary file 1 — Supplementary materials and methods [file CJP2-3-155-s007.docx]

**Supplementary Materials and Methods**

**Immunohistochemistry**

Paraffin-embedded colorectal cancer sections and matched adjacent normal sections were deparaffinised and heat-induced epitope retrieval (HIER) was performed with incubation with Novocastra Epitope Retrieval Solution pH8 (Leica) at 97°C for 40 minutes. Slides were then washed twice in diluted wash buffer (Dako) before treatment with dual endogenous enzyme inhibitor (component of Dako Envision Detection System peroxidase/DAB, Rabbit/Mouse kit, K406511-1). Slides were then washed twice as above. Non-specific binding was blocked with 1% normal goat serum (NGS) diluted in antibody diluent (Dako S0809) for 30 min. Primary mouse anti-human EPCR antibody (R&D Systems, clone 304519) was then added at 6.7μg/ml, diluted with antibody diluent, and incubated overnight at 4°C. Slides were then washed three times with wash buffer and incubated for 30 minutes with polyclonal anti-mouse secondary antibody (Dako, component of Envision Detection System), then washed three times as above. Peroxidase activity was revealed by adding diaminobenzidine (DAB – component of Dako Envision Detection System) for 5 minutes. The slides were counterstained with haematoxylin (100%, for 40 seconds) and were then washed under warm running tap water for 2 minutes. Slides were dehydrated in 70% ethanol for 2 minutes, then 100% industrial methylated spirit (IMS) for 2 minutes, and were then added to Histoclear for 2 hours (Fisher Chemical). Coverslips were mounted using DPX (National Diagnostics), and were left to dry overnight. Guidance on antibody optimisation was provided by a histopathologist (PT). Controls that omitted the primary antibody were also performed to rule out non-specific binding of the secondary antibody. For PAR1 IHC the same protocol was used with a primary antibody that targeted PAR1 (clone N2-11 Novus Biologicals), at 10 μg/ml. To detect EPCR and tryptase-positive mast cells in human lung sections, 10 nM citrate buffer was used for antigen retrieval on 4 µm sequential paraffin sections, followed by immunostaining with mouse anti-human EPCR monoclonal antibody (R&D systems as above, 10 μg/ml) and mouse anti-human tryptase (clone AA1, 0.1 µg/mL, DAKO).

Slides were imaged on a Vectra 2.0 (Perkin Elmer) system using custom imaging algorithms developed on Inform software (Perkin Elmer). Analysis of slides was performed on Inform software, using trained tissue and cell segmentation algorithms that were validated by a histopathologist (PT). Staining intensities were determined on a per cell basis, and H-scores were created by the Inform software. Tumour region and Stroma region H-scores across all slides were collated and compared using the unpaired T Test, after the normality of distributions were confirmed using the Anderson Darling test.

COIN study slides were stained for EPCR at the HBRC biobank using a Bondmax Autostainer. The primary antibody (R&D systems, clone 304519) incubation time was 10 minutes at a dilution of 1:200. Antigen retrieval was performed at pH9. Stained slides were scanned using a Leica SCN400 slide scanner. Scanned slides were analysed on Definiens Tissue Studio software. Tumour regions of each slide were manually identified, and trained segmentation algorithms were used to separate epithelium and stromal regions. Quantification of EPCR staining was performed on a regional basis, with the software quantifying the percentage of pixels that had strong staining, moderate staining, weak staining or no staining in each area. These data were used by the software to create a percentage score for each region in each slide.

**Cell culture**

Human colorectal cell lines (HT29, HCT116, Colo320, SW480, and RKO) were cultured in DMEM containing 10% fetal bovine serum (FBS). The AA/C1 adenoma line and its derivative AA/C1/SB/10C were obtained from Chris Paraskeva (36) and were cultured in DMEM containing 20% FBS, 100U/ml Penicillin, 100 μg/ml Streptomycin, 1ug/ml hydrocortisone (Sigma) and 0.2U/ml insulin (Sigma). The human mast cell (MC) line HMC-1.1 (V560G) (44) was cultured in IMDM with 10% FBS. Human lung mast cells (HLMCs) were isolated from healthy lung obtained at surgery for carcinoma using anti-CD117-coated Dynabeads to ∼99% purity (45). HLMCs were cultured in DMEM supplemented with 10% FCS, and cytokines (100 ng/ml SCF, 50 ng/ml IL-6, and 10 ng/ml IL-10) as described previously (46).

**Flow cytometry**

Colorectal cell lines and adenomas were stained with anti-human EPCR-allophycocyanin, eBioscience, clone RCR-227, 5 μl per test) or isotype control (Rat IgG1, κ allophycocyanin, eBioscience, clone eBRG1) and analysed on an Accuri C6 (BD Biosciences) or LSR II (BD Biosciences) flow cytometer, and data were analysed using FlowJo. HMC-1 cells and HLMCs were stained with 2E9 antibody (1 µg/mL) (15) which recognises EPCR or with IgM isotype control (Sigma), followed by FITC-anti-mouse IgM (Sigma) and analysed on a FACSCanto (BD Biosciences, Oxford, UK).

**Sigma MISSION shRNA clones**

The five EPCR shRNA clones for knockdown studies were Sigma MISSION lentiviral pLKO.1-puro transduction particles (details in the table below); a control shRNA clone was Sigma MISSION pLKO.1-puro Control high titre transduction particles- SCH001H.

|  | TRC number | Clone ID | Sequence |
| --- | --- | --- | --- |
| 1 | TRCN0000061378 | NM_006404.3-253s1c1 | CCGGTGGCCTCCAAAGACTTCATATCTCGAGATATGAAGTCTTTGGAGGCCATTTTTG |
| 2 | TRCN0000061382 | NM_006404.3-706s1c1 | CCGGTCGGTATGAACTGCGGGAATTCTCGAGAATTCCCGCAGTTCATACCGATTTTTG |
| 3 | TRCN0000300553 | NM_006404.3-679s21c1 | CCGGGCAGCAGCTCAATGCCTACAACTCGAGTTGTAGGCATTGAGCTGCTGCTTTTTG |
| 4 | TRCN0000369969 | NM_006404.3-1123s21c1 | CCGGTTTGCTGAATTAGTCTGATAACTCGAGTTATCAGACTAATTCAGCAAATTTTTG |
| 5 | TRCN0000061379 | NM_006404.3-743s1c1 | CCGGGTGCAGTATGTGCAGAAACATCTCGAGATGTTTCTGCACATACTGCACTTTTTG |

**Western blotting**

Activated Protein C was added for 2 mins, 5 mins and 10 mins in the presence/absence of EPCR function blocking antibodies (clone RCR-252, Novus Biologicals) at a concentration of 180nM to confluent HCT116 cells following 48 hours of serum starvation. Following this, cells were lysed with 10% SDS containing phosphatase inhibitor (Phosphatase Inhibitor Cocktail II, Sigma), and lysates separated by SDS-PAGE gel electrophoresis. Samples were transferred to PVDF membrane, blocked with 3% BSA in TBST, then incubated overnight at 4°C with primary anti-ERK or anti-pERK primary antibodies (New England Biolabs PhosphoPlus (Thr202/Tyr204) Antibody Duet (4370S + 4695S)), prior to anti-rabbit HRP conjugated secondary antibody addition. Blots were developed using an ECL detection kit (Biological Industries) and imaged using the CHEMIDOC system (Biorad), with image analysis using Image Lab 4.1 (Biorad).

Western blotting was also performed to determine whether PC and APC were present in culture media (DMEM) or HCT116 cell lysate or supernatant. Exogenous APC (Sigma), was used as a positive control in this analysis. The western blotting protocol was as described above, except that a protein C-specific primary antibody (clone NBP1-58065, Novus Biologicals) was used. This antibody detected both PC and APC, as it targeted the light chain of PC (PC comprises a light and heavy chain) from amino acids 1 to 236, which is found in both PC and the activated form APC.

**Microarray**

Optimal concentrations and treatment durations were determined by assessing APC-induced expression changes of 4 genes (NFkB2, PCNA, BCL2A1, EFNA1, which were chosen based on previous findings in endothelium (5)) using qRT-PCR (probes from Invitrogen)**.** A concentration of 180nM for 24 hours was shown to be associated with maximal gene expression change. Serum starvation was carried out for 48 hours prior to APC addition at 180nM for 24 hours. The purity and concentration of extracted RNA was validated spectrophotometrically before microarray analyses.

qPCR Taqman probe IDs (Invitrogen):

| **Genes** | **Probe IDs** | **NCBI reference Sequence** |
| --- | --- | --- |
| NFkB2 | Hs01028901_g1 | NM_001077494.2 |
| PCNA | Hs00427214_g1 | NM_002592.2 |
| BCL2A1 | Hs00187845_m1 | NM_004049 |
| EFNA1 | Hs00358886_m1 | NM_004428.2 |

**MTT assays**

HCT116 and HT29 shRNA EPCR knockdown cells were cultured in DMEM with 10% FCS. After 48 hours of incubation, chemotherapy drugs were added to the plates (either 5-Fluorouracil (5FU) or epirubicin) in concentrations determined by serial dilution (5FU – 0-256 µM, epirubicin – 0-8 µM). After a further 48 hours of incubation, MTT (3-(4,5-[Di](http://en.wikipedia.org/wiki/Di-)[methyl](http://en.wikipedia.org/wiki/Methyl)[thiazol](http://en.wikipedia.org/wiki/Thiazole)-2-yl)-2,5-di[phenyl](http://en.wikipedia.org/wiki/Phenyl)tetrazolium bromide) was added to each well and after 2 hours cells were lysed with DMSO and absorbance was determined at 490nm for 0.1 seconds using a Victor plate reader. Colour intensity was normalised against untreated controls.

**BrdU assays**

Assays were performed using the BrdU (bromodeoxyuridine) cell proliferation ELISA kit (Roche), as per the manufacturer’s instructions. Cells were plated and treated with epirubicin or 5FU as above.

**Migration assays**

70-80% confluent EPCR-perturbed and control HCT116 and HT29 cells were serum-starved by incubation in serum-free medium (DMEM) for 24 hours. Serum-starved cells were added to a Transwell insert (Corning) and migration towards DMEM containing 10% FCS was measured following 3 - 48 hours incubation. The cells on the lower side of the Transwell insert were fixed with 30% methanol and stained with 2% crystal violet for 2 hours at 4°C. The Transwell inserts were removed and the upper side of the membrane was cleaned with a cotton bud to remove the non-migrated cells. The inserts were photographed with a light microscope and camera in 5 set regions (upper left, upper right, lower left, lower right and centre) using Axiovision software (Zeiss). Cells were counted and analysed using Excel (Microsoft Corp). The Mann-Whitney U test was used to compare migration in control versus knockdown lines.

**Invasion assays**

70-80% confluent EPCR-perturbed and control HCT116 and HT29 cells were serum-starved as with the migration assays. Cells were suspended in serum-free DMEM and added to a QCM cell invasion assay insert (Millipore). The assay was performed as per the manufacturer’s instructions.

**COIN clinical data**

Data received included treatment arm (chemotherapy or chemotherapy plus cetuximab), demographic details (including age and sex), and clinical data including WHO performance status, number of metastases, timing of metastases, site of primary (within colorectum), serum carcinoembryonic antigen (CEA), microsatellite status and mutation status for key genes including KRAS, BRAF, NRAS and PI3K. Survival data included 12-week response, best response, death, censored time to death, progression and censored time to progression (PFS time).
