# Supplementary material for: Endothelial protein C receptor is overexpressed in colorectal cancer as a result of amplification and hypomethylation of chromosome 20q
Source: J Pathol Clin Res. 2017 Jul 14;3(3):155–70. doi: 10.1002/cjp2.70 (PMC5527318; doi:10.1002/cjp2.70)
Supplement: Supplementary file 2 — Supplementary figure legends [file CJP2-3-155-s006.docx]

**Supplementary Figure Legends**

**Figure S1.** Validation of EPCR antibody. (A) IHC staining of wild-type HCT116 cells with anti-EPCR antibody. (B) IHC staining of EPCR shRNA knockdown HCT116 cells with anti-EPCR antibody. (C) Positive staining of endothelium.

**Figure S2.** EPCR expression in mast cells. (A) Flow cytometry demonstrating EPCR protein expression on mast cells. (B) Flow cytometry data are presented for the HMC-1 cell line and human lung mast cells (HLMCs) as percentage of 2E9+ cells of the total cell population using the Overton method ([47](#_ENREF_44))(HLMC n=3). (C-D) Sequential sections demonstrating co-localisation of ECPR to tryptase positive mast cells within airway tissue (black arrows ×200 magnification). HM

**Figure S3.** Expression of EPCR on colorectal cancer cell lines. Expression of EPCR on (A) Colo320, (B) RKO, (C) SW480, (D) HT29, and (E) HCT116 cells by flow cytometry. Expression of EPCR on (F) AA/C1 non-tumourigenic adenoma cells or (G) the tumourigenic AA/C1/10C derivative. Isotype control staining is shown in grey and EPCR staining in black.

**Figure S4.** Detection of EPCR-associated proteins in CRC. (A) Detection of Protein C in HCT116 cell lysate. The anti-Protein C antibody detects both Protein C (62 KDa) and Activated Protein C (APC, 21 KDa). Exogenous APC was used as a positive control. A faint band was detected in the 60-65KDa range in the HCT116 lysate, suggestive of the presence of Protein C. (B) and (C) PAR1 immunohistochemistry in (B) CRC and (C) normal colon.

**Figure S5.** Gene set enrichment analyses. Enrichment plots for gene sets: Nagashima EGF signalling up (A), Zwang EGF persistently up (B), Amit EGF response 40 Hela (C), Uzonyi response to leukotriene and thrombin (D). Lower panels – all genes ranked in order of most overexpression after APC treatment to most underexpression. 0 represents equal expression after treatment. Middle panels - the black vertical bars represent genes from the relevant gene set. Top panels - the green line represents the running enrichment score for the gene set as the analysis walks down the ranked list, increasing the running-sum statistic when a gene is in a gene set and decreasing it when it is not. As the peaks of these green lines are towards the overexpression side of the gene list, these gene sets are highly enriched.
