# Supplementary material for: Endothelial protein C receptor is overexpressed in colorectal cancer as a result of amplification and hypomethylation of chromosome 20q
Source: J Pathol Clin Res. 2017 Jul 14;3(3):155–70. doi: 10.1002/cjp2.70 (PMC5527318; doi:10.1002/cjp2.70)
Supplement: Supplementary file 8 — Table S1. Clinico‐pathological data for the local colorectal cancer cohort [file CJP2-3-155-s008.pdf]

Table S1. Clinico-pathological data for the local colorectal cancer cohort.

Patient ID	Age	Gender	Tumour site	Pathological stage (pMx unless otherwise stated)
1514	72	Female	Sigmoid colon	pT4 pN1
1515	73	Female	Sigmoid colon	pT3 pN0
1516	84	Female	Caecum	pT4 pN0
1517	57	Female	Caecum	pT2 pN1
1518	68	Female	Rectosigmoid	pT3 pN1 pM1
1519	72	Female	Caecum	pT3 pN2
1575	81	Female	Sigmoid colon	pT3 pN0
1603	83	Female	Ascending colon	pT3 pN0
1609	50	Male	Sigmoid colon	pT2 pN0
1634	84	Male	Sigmoid colon	pT4 pN2
1637	64	Male	Caecum	pT3 pN0
1700	76	Male	Caecum	pT4 pN2
1701	75	Male	Rectum	pT3 pN1
1702	55	Male	Sigmoid colon	pT4 pN0
1705	53	Male	Splenic flexure and sigmoid colon	pT4 pN2
1707	75	Male	Sigmoid colon	pT3 pN0
1713	31	Male	N/A	PT4 pN1
1813	72	Male	Caecum	pT4 pN1
1977	66	Female	Caecum	pT2 pN0
1980	67	Female	Sigmoid colon	pT2 pN1
1982	80	Male	Rectum	pT3 pN0
1987	64	Male	Rectum	pT3 pN1
1988	83	Female	Caecum	pT4 pN1
1990	59	Female	Rectum	pT2 pN0
1997	82	Female	Caecum	pT2 pN0
2007	68	Male	Rectum	pT3 pN1
2024	84	Female	Rectum	pT3 pN1
2042	61	Male	Rectosigmoid	pT4 pN2
2103	62	Male	Caecum	pT4 pN2
2276	51	Male	Rectosigmoid	pT3 pN0
