# Supplementary material for: Endothelial protein C receptor is overexpressed in colorectal cancer as a result of amplification and hypomethylation of chromosome 20q
Source: J Pathol Clin Res. 2017 Jul 14;3(3):155–70. doi: 10.1002/cjp2.70 (PMC5527318; doi:10.1002/cjp2.70)
Supplement: Supplementary file 9 — Table S2. Clinico‐pathological data for the COIN trial colorectal cancer cohort [file CJP2-3-155-s009.pdf]

Table S2. Clinico-pathological data for the COIN trial colorectal cancer cohort.

**Characteristic**	**Summary statistics**							
	EPCR epithelium high		EPCR epithelium low		EPCR stroma high		EPCR stroma low	
	Mean	SD	Mean	SD	Mean	SD	Mean	SD
Age at randomisation (years)	62.45	9.65	61.44	10.34	62.32	10.28	61.57	9.74
	N	Percentage	N	Percentage	N	Percentage	N	Percentage
Number of patients	76		77		76		77	
Received cetuximab as part of COI	38	50%	44	57%	38	50%	44	57%
Female	27	36%	34	44%	28	37%	33	43%
WHO performance status 0	32	42%	32	42%	28	37%	36	47%
WHO performance status 1	39	51%	41	53%	44	58%	36	47%
WHO performance status 2	5	7%	4	5%	4	5%	5	6%
Site of primary tumour:								
Left colon	10	13%	13	17%	9	12%	14	18%
Right colon	30	39%	30	39%	36	47%	24	31%
Rectum	17	22%	10	13%	13	17%	14	18%
Sigmoid colon	7	9%	16	21%	9	12%	14	18%
Rectosigmoid junction	10	13%	5	6%	9	12%	6	8%
Transverse colon	0	0%	3	4%	0	0%	3	4%
Other	2	3%	0	0%	0	0%	2	3%
KRAS status:								
Wild-type	39	51%	34	44%	41	54%	32	42%
Mutation	33	43%	36	47%	33	43%	36	47%
Test failure	1	1%	1	1%	0	0%	2	3%
Not tested	3	4%	6	8%	2	3%	7	9%
MSI status								
Stable	55	72%	48	62%	53	70%	50	65%
MSI	2	3%	2	3%	2	3%	2	3%
Uncertain	0	0%	3	4%	1	1%	2	3%
Test failure	4	5%	2	3%	4	5%	2	3%
Not tested	15	20%	22	29%	16	21%	21	27%
