# Supplementary material for: Endothelial protein C receptor is overexpressed in colorectal cancer as a result of amplification and hypomethylation of chromosome 20q
Source: J Pathol Clin Res. 2017 Jul 14;3(3):155–70. doi: 10.1002/cjp2.70 (PMC5527318; doi:10.1002/cjp2.70)
Supplement: Supplementary file 10 — Table S3. Genes differentially expressed with APC treatment of HCT116 cells (Bayes Factor >5). Log FC shows fold change (base 2) in APC treated cells versus controls. 0 therefore represents equal expression in the APC treated and control cells [file CJP2-3-155-s010.pdf]

Table S3. Genes differentially expressed with APC treatment of HCT116 cells (Bayes Factor >5). Log FC shows fold change (base 2) in APC treated cells versus controls. 0 therefore represents equal expression in the APC treated and control cells.

**Gene ID**	**Gene name**	**logFC**	**P.Value**	**adj.P.Val**	**Bayes factor**
RPL13A	ribosomal protein L13a	0.729831	1.32E-09	5.04E-05	11.4789
RPS2	ribosomal protein S2	0.75432	2.37E-09	5.04E-05	11.07495
RPS21	ribosomal protein S21	0.64925	7.57E-09	8.69E-05	10.22977
RPL38	ribosomal protein L38	0.640211	8.88E-09	8.69E-05	10.10872
RPS20	ribosomal protein S20	0.56367	1.23E-08	9.05E-05	9.857144
RPL12	ribosomal protein L12	0.589346	1.61E-08	0.000105	9.647333
RPL26	ribosomal protein L26	0.527112	2.52E-08	0.000119	9.293517
RPS27	ribosomal protein S27 (metallopanstimulin 1)	0.569845	2.63E-08	0.000119	9.257807
EEF1A1	eukaryotic translation elongation factor 1 alpha 1	0.687543	3.33E-08	0.000125	9.067365
RPL23	ribosomal protein L23	0.691122	3.35E-08	0.000125	9.06284
RPL8	ribosomal protein L8	0.581833	3.41E-08	0.000125	9.047782
RPL35A	ribosomal protein L35a	0.614064	3.76E-08	0.00013	8.96871
RPL31	ribosomal protein L31	0.484977	7E-08	0.000188	8.453052
TACSTD2	tumor-associated calcium signal transducer 2	0.505105	8.88E-08	0.000226	8.251977
RPL37A	ribosomal protein L37a	0.457551	9.24E-08	0.000226	8.218734
UBC	ubiquitin C	0.505567	1.26E-07	0.000286	7.951056
RPL32	ribosomal protein L32	0.44282	1.27E-07	0.000286	7.948702
KITLG	KIT ligand	0.430458	1.36E-07	0.000289	7.885965
RPS18	ribosomal protein S18	0.430622	1.38E-07	0.000289	7.876527
DUT	dUTP pyrophosphatase	-0.4403	1.79E-07	0.000357	7.648652
GIPR	gastric inhibitory polypeptide receptor	0.665148	2.49E-07	0.000421	7.360759
RPS8	ribosomal protein S8	0.49689	2.51E-07	0.000421	7.35272
RPL7A	ribosomal protein L7a	0.431458	2.9E-07	0.00046	7.224366
RPS13	ribosomal protein S13	0.366457	4.07E-07	0.00052	6.922628
CLK1	CDC-like kinase 1	-0.38066	4.25E-07	0.000532	6.882765
RPL17	ribosomal protein L17	0.377933	4.57E-07	0.000555	6.818574
ING3	inhibitor of growth family, member 3	0.367639	5.26E-07	0.000596	6.692568
RPL3	ribosomal protein L3	0.446173	6.22E-07	0.000629	6.540546
RPS7	ribosomal protein S7	0.413569	8.01E-07	0.000677	6.309131
SLC1A3	solute carrier family 1 (glial high affinity glutamate transporter), member 3	0.36994	9.43E-07	0.000693	6.159936
LCN2	lipocalin 2 (oncogene 24p3)	0.483695	9.43E-07	0.000693	6.159605
MT2A	metallothionein 2A	0.448027	9.86E-07	0.000695	6.118647

RPL10A	ribosomal protein L10a	0.373973	9.91E-07	0.000695	6.113851
ANXA2	annexin A2	0.352575	1.02E-06	0.000695	6.089494
C10orf10	chromosome 10 open reading frame 10	0.396982	1.07E-06	0.000716	6.040645
HNRPH2	heterogeneous nuclear ribonucleoprotein H2 (H')	0.348645	1.17E-06	0.000757	5.958679
ARHGEF15	Rho guanine nucleotide exchange factor (GEF) 15	0.451955	1.24E-06	0.000776	5.910181
RPL36A	ribosomal protein L36a	0.459017	1.27E-06	0.000784	5.886451
RPS16	ribosomal protein S16	0.587827	1.33E-06	0.000806	5.841946
RPS3A	ribosomal protein S3A	0.38013	1.42E-06	0.000822	5.782664
RPL34	ribosomal protein L34	0.401456	1.5E-06	0.000845	5.732861
ATF6	activating transcription factor 6	0.330841	1.52E-06	0.000846	5.71839
FAU	Finkel-Biskis-Reilly murine sarcoma virus (FBR-MuSV) ubiquitously expressed (fox derived); ribosomal protein S30	0.402734	1.54E-06	0.000846	5.70528
BRP44L	brain protein 44-like	0.389064	1.68E-06	0.000881	5.624935
RPL13	ribosomal protein L13	0.526131	1.85E-06	0.000946	5.534252
MIF	macrophage migration inhibitory factor (glycosylation-inhibiting factor)	0.379488	1.85E-06	0.000946	5.533545
EIF4A3	eukaryotic translation initiation factor 4A, isoform 3	-0.33887	1.92E-06	0.000947	5.501359
DHTKD1	dehydrogenase E1 and transketolase domain containing 1	0.364546	1.94E-06	0.000951	5.489604
FAM49B	family with sequence similarity 49, member B	0.396678	1.97E-06	0.000955	5.477903
RPS26	ribosomal protein S26	0.454294	2.2E-06	0.001032	5.372796
RPL30	ribosomal protein L30	0.362797	2.21E-06	0.001032	5.367476
HRASLS5	HRAS-like suppressor family, member 5	0.456289	2.25E-06	0.001032	5.351502
TAPBP	TAP binding protein (tapasin)	0.312637	2.26E-06	0.001032	5.349157
CNOT7	CCR4-NOT transcription complex, subunit 7	0.352376	2.27E-06	0.001032	5.344529
RASD1	RAS, dexamethasone-induced 1	0.525178	2.31E-06	0.00104	5.326405
FKSG30	actin-like protein	0.315158	2.43E-06	0.001081	5.279509
SSBP4	single stranded DNA binding protein 4	-0.36137	2.67E-06	0.001143	5.192159
SPATA22	spermatogenesis associated 22	0.446524	2.86E-06	0.001206	5.125614
RPLP0	ribosomal protein, large,	0.376217	2.87E-06	0.001206	5.121352

	P0				
DTL	denticleless homolog (Drosophila)	-0.32126	2.91E-06	0.001212	5.10953
RPL10	ribosomal protein L10	0.320072	2.97E-06	0.001219	5.091869
RPS19	ribosomal protein S19	0.349232	2.97E-06	0.001219	5.090609
GJB3	gap junction protein, beta 3, 31kDa (connexin 31)	0.307522	3E-06	0.001225	5.08002
RPS12	ribosomal protein S12	0.319493	3.07E-06	0.001242	5.059895
HMBS	hydroxymethylbilane synthase	-0.31278	3.12E-06	0.001242	5.044439
DUSP1	dual specificity phosphatase 1	0.395831	3.12E-06	0.001242	5.043594
ATAD4	ATPase family, AAA domain containing 4	0.375962	3.15E-06	0.001242	5.034235
TES	testis derived transcript (3 LIM domains)	0.428908	3.27E-06	0.001244	5.000888
